# Sustainable Employability of People with Limited Capability for Work: The Participatory Development and Validation of a Questionnaire

**DOI:** 10.1007/s10926-024-10191-1

**Published:** 2024-05-20

**Authors:** S. R. Hiemstra, B. P. I. Fleuren, A. de Jonge, J. Naaldenberg, L. Vaandrager

**Affiliations:** 1 Health & Society, Wageningen University & Research, Wageningen, The Netherlands; 2 Faculty of Psychology and Neuroscience, Maastricht University, Maastricht, The Netherlands; 3 Ministry of the Interior and Kingdom Relations, Organisation and Personnel, Binnenwerk, The Hague, The Netherlands; 4 Department of Primary and Community Care, Radboud University Medical Center, Nijmegen, The Netherlands

**Keywords:** Sustainable employability, Limited capability for work, Disability, Work-related sense of coherence, Person-job fit

## Abstract

**Purpose:**

Understanding sustainable employability (SE) of people with limited capability for work (LCW) due to physical or mental disability is crucial for the sustainable participation of this target group. Therefore, adequate measurement instruments for SE are needed. This study aims to validate a questionnaire to measure SE among people with LCW using a participatory approach, including person-job fit (PJ fit) and work-related sense of coherence (Work-SoC).

**Methods:**

Existing scales for the main concepts were tested and adapted for face validity via cognitive interviews (*n* = 6), with the involvement of a co-researcher with LCW in the research team. Next, the questionnaire was administered among people with LCW (*n* = 248) to assess its factor structure (Confirmatory Factor Analysis) and reliability (Cronbach’s alpha).

**Results:**

Analysis of the cognitive interviews identified problems with clarity and readability of items, instructions and response categories of used (existing) scales. The main adjustments concerned the shortening of text length, the usage of familiar language and examples, and the addition of an introduction game. Most of the adapted SE indicator scales showed an overall good fit and acceptable-to-good internal reliability. The overall SE model had an overall good fit, and excluding ‘*internal employability’* further improved this fit. PJ fit and Work-SoC had an acceptable/good model fit and internal consistency.

**Conclusion:**

The participatory validation process resulted in a validated and comprehensive questionnaire to measure SE, PJ fit and Work-SoC among people with LCW, which enables research into the development of their SE. This questionnaire can be utilised to contribute to a more inclusive labour market.

**Supplementary Information:**

The online version contains supplementary material available at 10.1007/s10926-024-10191-1.

## Introduction

For people with limited capability for work (LCW) due to physical or mental disability, having a job contributes to health, well-being and personal growth, and to important values such as inclusion in society and full and equal enjoyment of all human rights. Many countries, including the Netherlands, ratified the United Nations Convention on the Rights of Persons with Disabilities and aim to stimulate the inclusion of people with disability in the labour market [1]. However, despite formal policies, employment rates lag behind and part time, temporary contracts are the norm [2, 3]. In the Netherlands, approximately 1.8 million people experience LCW [4]. Around 50% of the people with capability for work are working, of which only 60% are still in employment after one year [5]. This is alarming, since many people with LCW are able to work and eager to do so [6].

Research on sustainable employability (SE) of people with LCW is crucial for a movement towards more sustainable participation for all. SE refers to an individual’s chance to find and maintain work [7, 8]. Despite its importance, research on SE has not yet specifically targeted people with LCW, which consists of a heterogeneous group of individuals who experience functional limitations (such as limitations in cognition, communication, social interactions, etc.) which may overlap between different disabilities. People with LCW may experience a combination of functional limitations, and their severity varies from person to person. These functional limitations limit the ability of this target group to engage in (regular) paid work without additional support or accommodations. Studies on SE that do exist for people with LCW typically consider a small array of indicators (e.g. job satisfaction, work motivation and work engagement [9, 10]) and do not allow for a comprehensive understanding of SE, as this construct is multidimensional in nature.

While integrative measurement instruments for SE are available for a general population [11, 12], recent calls stress the importance of developing instruments that are specifically designed for people with LCW [13]. Accessible questionnaires with clear instructions and items that are tailored to the abilities and experience of people with LCW are crucial for participation and valid results. Research conducted with similar groups showed that the experiential knowledge of employees with LCW can contribute to the adaptation and validation of questionnaires [14, 15]. With appropriate, adapted, instruments, we will gain a better understanding of how to create opportunities for sustainable employment for this large, important and diverse group of people.

This study aims to develop and validate a questionnaire to measure the SE for people with LCW. Specifically, we draw on the validated measurement instrument for SE by Fleuren et al. [12] as used among a general working population. Additionally, the questionnaire includes person-job fit (PJ fit) and work-related sense of coherence (Work-SoC) as close antecedents of SE. This paper firstly presents a participatory adaptation process to develop a measurement instrument for SE that is suitable for people with LCW. To ensure face validity of the resulting instrument, we use cognitive interviews and include a co-researcher with LCW in our research team. Secondly, we assess the dimensionality and validity of the tailored questionnaire using confirmatory factor analysis on self-reported questionnaire data of a large sample of people with LCW. Specifically, we include people with LCW to whom the Dutch Participation act applies and who work for a governmental organisation that facilitates a diverse range of paid jobs for this target group. With this approach, the present article provides a validated questionnaire to assess the SE of people with LCW, thereby enabling more profound research on their SE. Moreover, the current approach provides an extensive illustration of how existing instruments to measure complex constructs can be adapted to suit specific populations and settings.

## Sustainable Employability

In this paper, we define SE as follows: ‘*that an individual’s ability to function at work and in the labor market, or their ‘employability’, is not negatively, and preferably positively affected by that individual’s employment over time’* [8]. This definition operationalises SE as an individual and formative construct, with a set of complementary indicators of functioning in terms of health, well-being and competence that should ideally be considered over time. Indicators in the health domain include perceived health, work ability (the ability to work given personal health status) and measures of fatigue (typically captured as the need for recovery and/or fatigue). These indicators are complemented by job satisfaction and motivation to work to cover occupational well-being, as well as perceived employability, skill gap and job performance as indicators of competence. Several other definitions of SE exist (e.g. [11, 16, 17]), but these definitions suffer from shortcomings that the current definition resolves (see [8] for an in-depth discussion).

Other approaches to SE offer theoretically relevant antecedent constructs that enrich our understanding of SE. The most cited approach to SE, the capability approach, is based on the notion that generating value through work is central to SE [11]. Its premise is that if workers can achieve value at work, they will be sustainably employable. This approach has raised considerable criticism because it simultaneously treats and defines SE as a characteristic of the job and the employee [18]. To gain a better understanding of individual SE and how this is affected by work, we need instruments that enable us to disentangle the value of work from an individual’s SE. The salutogenic perspective offers means to do so, as it underscores the importance of an individual’s experience of work as meaningful, manageable and comprehensible—i.e. work-related sense of coherence (Work-SoC) [19]. Strong Work-SoC has been associated with well-being (e.g. fatigue) and positive work outcomes (e.g. work involvement) in normal work populations [19, 20]. As such, we consider Work-SoC as a valuable way of operationalizing the idea of achieving value in work in the context of SE, acknowledging it as close antecedent factor of individual SE.

The ability to generate value through work depends on the interaction between individuals and their work environment. Therefore, we expand the questionnaire also with person-job (PJ fit). PJ fit describes to what extent the abilities and characteristics of a person align with (the tasks of) their job. PJ fit consists of two dimensions. The demands-ability fit refers to extent to which an employee has the skills, knowledge and capabilities to accomplish the requirements of the job. The needs-supplies fit represents the extent to which work accommodates an individual’s needs [20]. A strong PJ fit is positively related to SE indicators in general work populations (e.g. job satisfaction and performance [20]) and has been used as an indicator of SE [16]. For people with LCW, an association is shown with exhaustion and work engagement [21], although instruments for regular work populations were used. Literature further indicates a relation with job satisfaction for this target group, although the evidence is limited [22]. By including PJ fit, we recognize its relevance for meaningful work experiences and individual SE.

## Methods

### Overview

The study consisted of two phases. The first phase developed and improved the face validity of the predefined questionnaire for SE using cognitive interviews and a participatory approach. The second phase involved administering the adapted questionnaire to people with LCW to assess its reliability and factor structure. Informed consent of participants was asked prior to research activities, and the study was approved by the Wageningen Scientific Ethical committee.

#### Setting

This study was part of a larger longitudinal study on the effects of work in natural environments on the health and SE of people with LCW in the Netherlands. This study was carried out at a central governmental organisation in the Netherlands that works closely with other governmental departments (e.g. the Dutch State Forestry Service or the Dutch Tax Administration) to provide paid work for people with LCW, who meet the LCW criteria of the Dutch Participation Act. Employees carry out work in, e.g. forestry, facility management or archive editing and receive practical and social support from their work supervisor. The organisation aims for job security for people with LCW, providing the opportunity for permanent employment after an initial probationary period.

#### Initial Questionnaire

The initial questionnaire contained 56 items, including scales regarding SE, PJ fit and Work-SoC (41 items). When possible, the authors used existing and validated scales for the main concepts. If the scales were not available in Dutch, they were translated forward and backward to maintain their original meaning [23]. The scales are displayed in Table 1. In Phase I, the full questionnaire (including informed consent, socio-demographic factors and work-related control variables) was tested and adjusted where necessary, because complex introductions or too many (difficult) items can lead to response burden. Response burden may affect the responses of participants, influencing the validity of the main scales [24]. The items capturing SE, PJ fit and Work-SoC can be found in Supplement A.

**Table 1:** Scales used for the initial questionnaire

Construct measured	Items	Scale used	Sample item
Sustainable employability			
Perceived health status	1	Medical outcomes study 36-item short form health survey (MOS SF-36) [25]	‘In general, would you say your health is: (1) excellent; (2) very good; (3) good; (4) fair; (5) poor’
Need for Recovery	6	Need for recovery scale from the Dutch Questionnaire for Experience and Evaluation of Work 2.0 [26]	‘I find it difficult to relax at the end of the working day’; always/often/sometimes/never
Work ability	2	Self-constructed, adapted from the single item for perceived health status MOS SF-36	‘In general, I feel physically healthy enough for my job’ and ‘in general, I feel mentally healthy enough for my job’; 5-point Likert scale
Skill gap	3	Demands-abilities subscale of the person-job fit scale [27]	‘My abilities and training are a good fit with the requirements of my job’; 5-point Likert scale
Employability	4	External employability constructed by Janssens, Sels & Van den Brande (3 items) [28] and internal employability (self-constructed, 1 item), each measuring a different aspect of employability	‘I am confident that I would find another job if I started searching’ and ‘I am confident that I can keep my current job’; 5-point Likert scale
Performance	4	Adapted from the core task performance scale [12, 29]	‘I fulfil the responsibilities that are described in my job description’; 5-point Likert scale
Motivation	9	Utrecht work engagement scale (UWES-9) [30]*	‘At work, I feel bursting with energy’; never/a few times a year or less/once a month or less/a few times a month/once a week/a few times a week/every day
Job satisfaction	1	Derived from Roelen, Koopmans & Groothoff [31]	‘In general, I am satisfied with my job’; 7-point Likert scale
Work-related sense of coherence	9	Work-SoC scale [19], measuring comprehensibility (4 items), manageability (3 items) and meaningfulness (2 items) of current job	‘How do you personally find your current job and work situation….’ (item 1: ‘… manageable vs unmanageable’; item 2: ‘… structured vs unstructured’; item 4: ‘… easy to influence vs impossible to influence’); 7-point scale to appraise the self-score for the opposing continuums
Person-job fit	6	Person-job fit scale [27], measuring demands-abilities fit (3 items) and needs-supplies fit (3 items) with current job	‘The match is very good between the demands of my job and my personal skills’ and ‘The attributes that I look for in a job are fulfilled very well by my present job’; 5-point Likert scale

*Due to authors’ rights, adjusting items during this study was not permitted

### Phase I: Improving Face Validity and Comprehensibility

#### Study Design

Phase I consisted of a participatory approach involving active participation of people with LCW [32]. First, people with LCW participated in cognitive interviews [33] that aimed to systematically investigate and improve the clarity, comprehensibility and content validity of the questionnaires. During the cognitive interviews, participants provided feedback and identified potential improvements. This method has been used successfully with similar target groups before [14, 15]. Second, the research team included an employee with LCW as co-researcher (AdJ), following guidelines for inclusive research [34], who actively contributed to the interpretation of the cognitive interviews results and the design of the final questionnaire (e.g. item and instruction formulations). Here explicit roles included reflecting and offering a sounding board for the researchers, designing and writing for new questions and procedures and co-deciding on adjustments.

#### Participants

Six employees with LCW were included in the cognitive interviews. Participants were recruited using convenience sampling at one work site within the overall project. All participants were men, and their ages ranged from 18 to 33 years. Their educational level ranged from (special) primary education to post-secondary vocational education (see Table 2). All participants experienced LCW due to mental or physical health disabilities, among which mild intellectual disabilities, severe dyslexia and/or physical and mental problems. Although only male participants were included in this stage, they experienced different limitations and represented a broad spectrum of people with LCW. The co-researcher was female and provided input in the interpretation of the cognitive interview results.

**Table 2:** Demographics of participants in Phase I & II

	Phase I (*n* = 6)	Phase II (*n* = 248)
	Cognitive interviews	Construct validity
Age		
Mean (SD)	26,2 (*6,3*)	33,9 (12,4)
Range (min–max)	18–32	18–64
Gender		
Female	0	56
Male	6	187
Education*		
No education or (special needs) primary education	1	23
(Special needs) secondary education	0	41
Practical secondary education	1	41
Post-secondary vocational education	4	126
Higher professional education or University	0	10

*clustering is based on Dutch Education system

#### Procedure

**Fig. 1:**
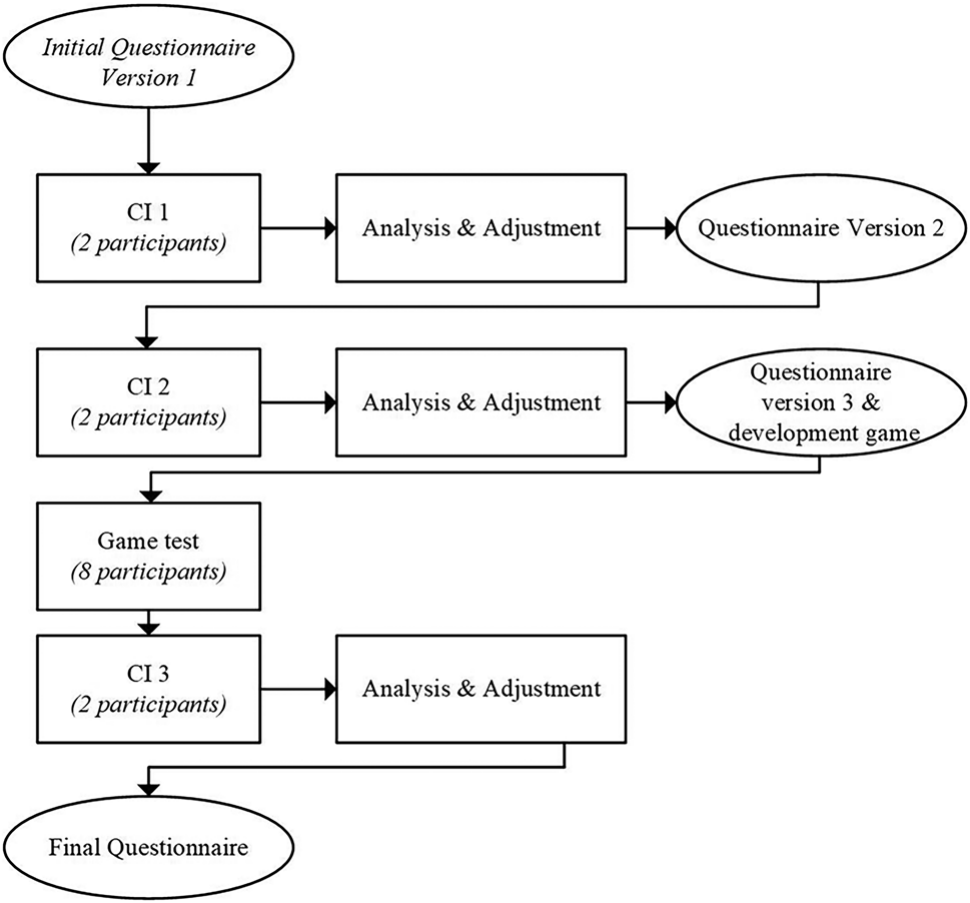
Process of Phase I

During the cognitive interviews, the principal investigator (SRH) asked individual participants to complete the questionnaire digitally on Microsoft Forms, to read the questions and their responses out loud and to explain how they decided to respond. This provided first insights about the comprehensibility of items. The principal researcher then asked additional probing questions, to identify both expected and unexpected challenges [14, 33]. The interview guide was based on the Question Appraisal System (QAS-99) by Willis [33] and contained instructions for the cognitive interviews, definitions of the intended meaning of items and probing questions. When problems were identified, participants were asked about potential improvements. Interviews lasted between 39 min and 1 h. Three rounds of two cognitive interviews each were conducted iteratively. The interviews were audio-recorded, and additional notes were made. The interviews were transcribed intelligent verbatim (see Fig. 1 for a visualisation of the process).

#### Analysis & Adjustment of Questionnaire

First, the principal investigator identified and clustered problems and suggestions for improvement. This process resulted in an overview containing the initial intended meanings of items, identified problems within the QAS-99 classification and participant suggestions for improvements. Subsequently, the co-researcher read the overview in silence, while the principal researcher was present for emerging questions. The co-researcher noted salient issues and additional suggestions for improvement.

After the analysis, the principal researcher and the co-researcher discussed the results and decided with consensus which suggestions, changes, additions and deletions would improve the questionnaire for people with LCW. Instances of doubt on improvements were recorded by the principal researcher. Finally, the adjusted concept questionnaire was discussed among the researchers (SRH, BPIF, LV) to check for scientific rigour and legal boundaries and to resolve instances of doubt.

### Phase II: Test for Construct Validity

#### Procedure & Participants

The adapted questionnaire was administered to employees of the governmental organisation that participated in the longitudinal study. The questionnaire was introduced to employees by their supervisors, who explained the purpose of the study to their team and facilitated the introduction game. After this, supervisors shared the questionnaire link with their employees, who could voluntarily decide whether to participate. Participants were asked to complete the questionnaire independently (if possible) during working hours. In total, 248 employees participated in the study. Their average age was 33.9 (*SD* = 12,40) years (*n* = 248). The majority of participants identified as male (*n* = 187), and attended post-secondary education (*n* = 126) (Table 2).

#### Analysis

To assess dimensionality and validity of the tailored questionnaire, confirmatory factor analysis (CFA) with Mplus 7 (Muthén & Muthén, Los Angeles) was applied (1) to test the factor structure of each multiple-item scale with three or more items for the indicators of SE and (2) to test the overall models for SE, PJ fit and Work-SoC. To test the overall model of SE, indicator scales of which a low score indicated high employability were reverse-coded to ensure that a high score would contribute positively to SE. Of interest in these analyses were model fit and factor loadings. Regarding model fit, the goodness-of-fit was based on the Comparative Fit Index (CFI) and the Tucker-Lewis Index (TLI) (≥ 0.90), the root mean square error of approximation (RSMEA) (≤ 0.08) and the assessment of factor loadings (≥ 0.3) [35]. The fit of the model was evaluated by the overall fit based on a majority of the fit indices. The weighted least squares mean and variance adjusted (WLSMV) estimator was used because inspection of the data suggested that the response distributions were not normal [36]. For completeness, we also estimated the internal consistency of the scales using Cronbach’s alpha, where α ≥ 0.7 indicates acceptable reliability.

## Results

### Phase I: Improving Face Validity and Comprehensibility

In total, 173 experienced problems were identified (Table 3). 144 adjustments were made to the questionnaire by modifying, adding or deleting an item, a response category or an instruction. These adjustments led to the final version of the questionnaire (57 items), including items for SE indicators (20), PJ fit (6), Work-SoC (9) and socio-demographic questions and work-related control variables (22). The total number of items increased due to additional socio-demographic and work-related control variables. The total number of items to measure SE, Work-SoC and PJ Fit decreased. Overall, there was a decrease in identified problems after each cognitive interview round. Most problems occurred in the QAS-99 components ‘clarity’, ‘reading’ and ‘response categories’. The results of Phase I for the QAS-99 categories are presented in Table 3, and the final items for SE, PJ Fit and Work-SoC are presented in Supplement A.

**Table 3:** Overview of identified problems and adjustments

	Version 1	Version 2	Version 3	Total
Number of cognitive interviews	2	2	2	6
Number of identified problems	83	62	26	173
Number of adjustments	66	61	17	144
Problems per QAS-99 component				
Clarity	28	16	7	51
Reading	21	20	6	47
Response categories	18	13	5	36
Assumptions	5	4	4	13
Instructions	3	3	2	8
Knowledge/memory	4	3	1	8
Sensitivity/bias	4	3	0	7
Other problems	1	1	1	3
Final version				
Number of total items	56	71	58	57
Socio-demographic & work-related	15	36	22	21
SE	26	20	20	20
Work-SoC	9	9	9	9
PJ Fit	6	6	6	6

*QAS-99* question appraisal system, *SE* sustainable employability, *Work-SoC* work-related sense of coherence, *PJ Fit* person-job fit

#### Clarity

Regarding clarity, 51 problems were identified, mostly due to difficult or unfamiliar words (such as the Dutch word ‘vervoering’/ ‘ecstasy’ from ‘Utrecht Work Engagement Scale—9’) or technical terms (like ‘production standard’). These issues were addressed by adding examples, using more familiar language, removing items, and experimenting with shorter/longer versions of the scales (which led to the use of ‘Utrecht Work Engagement Scale—3’ [37]).

#### Reading

Participants experienced 47 problems reading the items or interpreting these correctly, due to uncertainty about how or what to read and missing information. Examples include long introduction texts, missing information and items that start with one sentence followed by several statements, causing the cued context to be lost (i.e. with the items from the original Work-SoC questionnaire: ‘*I find my work …. Manageable/meaningful/predictable*’). Text length was adjusted, and complete sentences were added for each statement.

#### Response Categories

Regarding ‘response categories’, 36 problems occurred. Exemplary was the Work-SoC scale that originally contained nine items consisting of two opposite terms with a 7-point Likert scale in between. Participants were unsure whether the 7-point scale was supposed to represent a gradual shift from one term to the other. Additionally, other scales were scored using agreement-oriented 5-point Likert scales while yet others used 7-point scales or used temporal classifications for scoring, e.g. ‘once a week’. This confused participants and required them to seriously concentrate while completing the questionnaire. These problems were addressed by experimenting with new layouts and adjusting response categories when possible to a 5-point Likert scale ranging from ‘totally disagree’ to ‘totally agree’ with labels for each scale point in between.

#### Assumptions

Thirteen problems arose due to incorrect assumptions by the researchers about the context and work situation of participants. For example, one item addressed the extent to which the participant meets the performance standard of their job [29]. However, in the current work situation of participants, work was actually adjusted to individual capacity instead of a performance standard. In these cases, items were reframed to better suiting descriptions, e.g. *‘I do my work well’*.

#### Instructions

Problems with ‘instructions’ were identified eight times and included complexities in introductions, instructions or explanations. For example, when a subscale was longer than one page, the guiding text was repeated on the next page, causing confusion (‘we just completed these questions’). The problems were addressed by clustering subscales on one page or explicitly referring to a continuation of questions for a specific topic.

#### Knowledge and Memory

Eight problems occurred because participants did not know or had trouble remembering specific information. For example, recalling the specific month in which participants started working at their current job proved difficult. If possible, items that led to these errors were eliminated or cued with examples.

#### Sensitivity and Bias

Seven problems were identified with respect to sensitive language or content. Items or topics could make respondents uncomfortable or experience (negative) emotions, for example items regarding educational background. The answer possibility ‘I prefer not to say’ was added.

#### Questionnaire Administration Procedures

After the second cognitive interview round, it became evident that linguistic modifications couldn’t address challenges such as difficulty remembering dates or emotional responses. Based on suggestions from the cognitive interviews and the collaboration with the co-researcher, we decided to familiarise participants with these items before administering the questionnaire. To achieve this, we created an introductory game, as games can help people to reflect on challenging topics [34]. The game consists of a twelve-sided die with both challenging and easy questionnaire items. The game is designed as a team effort and is meant to be played in a team of employees with LCW. Employees take turns rolling the die and answer the questions themselves or ask a colleague to do so. Before administering the questionnaire, supervisors of these employees introduce the purpose of the study and facilitate the game. They guide the conversation and help to interpret the items.

To assess the game’s effectiveness, we invited the complete work-site team that provided the convenience sample for the cognitive interviews before the third cognitive interview round. This team (*n* = 8) comprised the work supervisor, four previous cognitive interview participants, two employees who did not yet participate in cognitive interviews and the co-researcher. The researcher was present and available for questions. Analysis of cognitive interview round 3 showed that participants recognised the items introduced by the game and that difficulties related to remembering answers and emotional charge had decreased. Consequently, the game was integrated into the questionnaire procedure of the final questionnaire.

### Phase 2: Validity Assessment Results

The results show a generally good fit for most of the scales for the SE indicators (Table 4), except for external employability (CFI = 0.95; TLI = 0.85; RMSEA = 0.303 [CI 90% 0.206–0.414]) and work engagement (CFI = 0.91; TLI = 0.74; RMSEA = 0.533 [CI 90% 0.432–0.642]). The RMSEA did not meet the cut-off criteria for any SE indicator scale. However, this failure to meet the cut-off criteria should be interpreted with caution, as RMSEA can falsely indicate a poor fit due to low degrees of freedom [38], which was the case in this study (Table 4). For all indicator scales, the factor loads for all items were adequate (Supplement B) and the internal reliability proved to be acceptable-to-good (α = 0.72–0.83).

**Table 4:** Results validity and reliability assessment of multiple-item indicators of SE, Work-SoC and PJ fit and the overall model for SE, including descriptive statistics of all SE indicators

	Mean (mdn)	SD	χ2	Df	CFI	TLI	RMSEA	RMSEA 90% CI	⍺
Indicators SE (N):									
Health (246)^a^	3.3 (3.0)	0.8							
Need for Recovery (237)	26.01 (22.2)	17.9	42.426*	9	0.928	0.940	0.141	0.106–0.179	0.82
Workability (246)^a^	4.1 (4.0)	0.7							0.72
External employability (248)	2.8 (3.0)	0.9	28.833*	1	0.951	0.852	0.303	0.206–0.414	0.75
Internal employability (248)^a^	4.3 (4.0)	0.7							
Performance (248)	4.3 (4.3)	0.5	14.044*	2	0.991	0.974	0.156	0.086–0.237	0.83
Motivation (247)^a^	4.3 (4.0)	0.7							
UWES-3 (246)**	4.9 (5.3)	1.3	70.994*	1	0.912	0.737	0.533	0.432–0.642	0.79
Job satisfaction (246)^a^	4.2 (4.0)	0.8							
Model SE:									
SE***^b^			424.008*	222	0.910	0.888	0.061	0.052–0.069	
SE***^b^ (without internal employability item)			2375.474*	253	0.931	0.915	0.054	0.044–0.063	
Antecedents									
Work-SoC (247)	3.9 (3.9)	0.5	59.261*	24	0.979	0.969	0.077	0.053–0.102	0.73
Person-Job Fit (247)	3.8 (3.8)	0.8	169.067*	8	0.963	0.930	0.286	0.249–0.324	0.90

^*a*^*indicator scale with* < *3 items*

^*b*^*formative construct, Cronbach’s alpha was not calculated*

*s*ignificant at p* < .05. **Factor variance was fixed to 1 to circumvent model saturation, ***ML estimator was used

The overall hypothesised SE model, based on the subscales for the indicators, was found to have a generally good fit (CFI = 0.91; TLI = 0.88; RSMEA = 0.061 [CI 90% 0.052–0.069]). Factor loadings proved to be adequate (Supplement B). However, the cognitive interviews suggested that in the specific context of this research, internal employability could rather be an indicator of organisational policy than the actual internal employability of the individual. An exploration of a competing model (Supplement B) revealed that the final questionnaire with the structure of the hypothesised factor and without the internal employability item provided a better and adequate model fit (CFI = 0.93; TLI = 0.92; RSMEA = 0.054 [CI 90% 0.044–0.063]).

Regarding the antecedents of SE, the results show an acceptable-to-good overall fit and internal consistency for both PJ fit (CFI = 0.96; TLI = 0.93; RMSEA = 0.286 [CI 90% 0.249–0.324], α = 0.90) and Work-SoC (CFI = 0.98; TLI = 0.97; RMSEA = 0.077 [CI 90% 0.053–0.102]; α = 0.73). Factor loadings were sufficient for all PJ fit and Work-SoC items (Supplement A).

## Discussion

This study aims to validate a questionnaire to measure SE, PJ fit and Work-SoC for people with LCW. The study was conducted at a central governmental organisation that works closely with governmental departments to provide paid work for people with LCW, who meet the LCW criteria of the Dutch Participation Act. First, the results show that the participatory validation process contributed to the improvement of the face validity of items for SE, PJ fit and Work-SoC for people with LCW, as demonstrated by the number and nature of adjustments. Second, the study demonstrates that the tailored scales for SE, PJ fit and Work-SoC are valid and reliable instruments to measure SE, PJ fit and Work-SoC for people with LCW.

The cognitive interview results show that face validity benefited most from improving the clarity of the questions, the answer categories, the accompanying instructions and the procedure. Based on the results and the cut-off scores of this study, the final questionnaire had good psychometric properties and can be used for future research on SE, PJ fit and Work-SoC for people with LCW. These results are in line with recent studies that developed other questionnaires for people with disability [14, 15]. Noteworthy is that the ‘Utrecht Work Engagement Scale—3’, which was not adjusted due to copyrights, appears to have the weakest fit results of the indicators examined for SE. However, previous studies on the Utrecht Work Engagement scales also showed weak goodness-of-fit statistics in general populations [39].

Regarding the Work-SoC scale, it is noticeable that factor loadings for item 4 (*λ* = 0.477) and item 9 (*λ* = 0.328) are relatively low. This suggests that the subscales for manageability (item 4) and comprehensibility (item 9) are not unidimensional. Based on our results, it is difficult to distinguish whether these findings are due to the specific target group or to dimensionality issues of the subscales. Studies in general working populations have also shown problems with other items and the dimensionality of subscales [40, 41].

The current study underlines the importance of clarity of questions, response options and instructions, and feasible procedures. Furthermore, the results show the importance of contexts for the validity of measurement scales [42], as illustrated by the item on internal employability in this study. This item was understood as intended and reflected the possibility of a participant to keep their job at their current employer. However, this seemed to be due to organisational policy rather than to reflect the employability of an individual. Removing this item led to a better model fit. While these findings are presented in the light of validity for people with LCW, one could argue that unambiguous measurement instruments, based on correct assumptions about target groups and their context, are beneficial for broader research populations.

### Strengths and Limitations

A major strength of this study is the participatory approach. Cognitive interviews and the cooperation with a co-researcher allowed for the systematic observation and discussion of problems in order to optimise the questionnaire and its procedures. In line with other participatory studies, the results showed that incorporating experiential knowledge positively impacted the suitability and validity of the questionnaire [14, 15, 43]. Additionally, the participatory approach evoked co-learning and empowerment processes, as is recognised in other inclusive research settings [44, 45]. For example, the co-researcher experienced opportunities to gain new skills and knowledge, and researchers became more aware of the context and implications of their activities. In addition, the iterative approach of this study allowed to adapt administration procedures with additional features, reducing the burden on participants and contributing to validity.

A first important limitation of our study is the specificity of the employment setting, which limits the generalisability of our results to different settings or professions. Second, we aimed to develop and validate a questionnaire on SE for people with LCW in general. Given the desired heterogeneity of our sample that matches the target population, it might be relevant to check if different subgroups (e.g. based on gender, age, education level or mental/physical condition(s)) show measurement invariance [46]. However, despite our relatively large sample size (i.e. *N* = 248; for this target population obtaining larger samples is challenging), subgroups became too small to perform adequate SEM with invariance tests in this study. Future studies should further explore if invariance exists, specifically by establishing the stability of the reflective measurement models of the scales used and the formative indicator-to-construct paths across subgroup categories. A third apparent limitation resides in the relatively small convenience sample consisting exclusively of males in Phase I. However, this sample was diverse regarding experienced disabilities and the co-researcher could supplement the female perspective. The method allowed for in-depth reflections, thereby ‘guiding “best informed” design decisions’ [47], and there were no indications that a lack of diversity in perspectives biased our findings. Nonetheless, future studies may want to include more (e.g. gender-) diverse samples. A fourth consideration is that, ideally, longitudinal studies should be conducted given SE’s longitudinal nature. As noted in a similar cross-sectional validation study on SE, the present cross-sectional study offers an important basis for doing so among people with LCW [12]. In sum, the aforementioned limitations emphasise the importance of replication studies among diverse settings, samples and timeframes. The questionnaire developed in the present study offers means to do so.

#### Implications for Occupational Science and Practice

The current study provides first a validated questionnaire to measure SE, PJ fit and Work-SoC for people with LCW. The study’s participatory approach is an example of how researchers can tailor questionnaires to specific target groups, in specific settings. This example highlights the importance of participatory collaboration in psychological research [48]. Second, the validated questionnaire enables researchers to measure SE across a diverse range of people with physical and mental disability, while acknowledging the antecedent importance of aligning work with individual needs, capacities and values [7, 16]. However, when this questionnaire is used in another country or setting, it is recommended to reapply cognitive interviews with diverse samples to make sure that face validity is not lost in translation.

Furthermore, the findings raise new questions about the measurement of Work-SoC for the target group and broader research populations. The study found low factor loadings for the comprehensibility and manageability dimensions. These have proven difficult to discriminate in general populations [40, 41]. However, specific items may have limited success in capturing comprehensibility and manageability for people with LCW. Further research is needed to explore the underlying factor structure of Work-SoC in different populations. Nevertheless, at this moment, the tailored Work-SoC scale serves as the best instrument to measure Work-SoC for people with LCW in a Dutch context.

Future studies demonstrating the broader validity of the questionnaire can contribute to further insight into what makes work ‘work’ for whom and when. This knowledge can improve the development and evaluation of supportive work environments for people with LCW, thereby contributing to sustainable work participation.

## Conclusion

The participatory validation process carried out within a governmental employment setting for people with LCW resulted in a validated and comprehensive questionnaire to measure SE, PJ fit and Work-SoC for this target group, which enables research into the development of their SE. This knowledge can be used to contribute to a more inclusive society with meaningful occupations for people with LCW.

## Supplementary Information

Below is the link to the electronic supplementary material.

Supplementary file1 (DOCX 49 KB)

Supplementary file2 (DOCX 116 KB)

## Data Availability

The data supporting the findings of this study are available upon reasonable request from the corresponding author. The data are not publicly available due to privacy or ethical restrictions.
