# Supplementary material for: Sustainable Employability of People with Limited Capability for Work: The Participatory Development and Validation of a Questionnaire
Source: J Occup Rehabil. 2024 May 20;35(1):105–15. doi: 10.1007/s10926-024-10191-1 (PMC11839800; doi:10.1007/s10926-024-10191-1)
Supplement: Supplementary file 1 — Supplementary file1 (DOCX 49 KB) [file 10926_2024_10191_MOESM1_ESM.docx]

# Supplementary information A

**Article title:** Sustainable Employability of People with Limited Capability for Work: the Participatory Development and Validation of a Questionnaire

**Journal:** Journal of Occupational Rehabilitation

**Authors:** S.R Hiemstra, B.P.I. Fleuren, A. de Jonge, J. Naaldenberg & L. Vaandrager

**Corresponding Author:** S.R. Hiemstra

**Introduction**

This electronic supplement provides the (tailored) items used in the ultimate questionnaire to measure SE, PJ-Fit and Work-SoC for people with Limited Capability for Work in Phase II of the study. We present them in the used order of items and in Dutch. Translated English items used to measure SE, PJ-Fit and Work-SoC in the final questionnaire can be found in Supplement B, where we present the tested factor models and factor loadings of individual items.

# Sustainable Employability

***Perceived Health***

Wat vind je, over het algemeen, van jouw gezondheid? *(Perceived health)*

| Helemaal Oneens | Oneens | Neutraal | Eens | Helemaal Eens |
| --- | --- | --- | --- | --- |
| 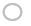 | 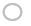 | 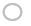 | 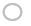 | 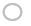 |

***Workability***

Ik voel mij over het algemeen lichamelijk gezond genoeg voor mijn werk. *(Workability 1)*

*Vul het best passende antwoord in.*

| Helemaal Oneens | Oneens | Neutraal | Eens | Helemaal Eens |
| --- | --- | --- | --- | --- |
| 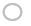 | 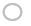 | 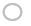 | 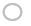 | 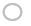 |

Ik voel mij over het algemeen mentaal gezond genoeg voor mijn werk. *(Workability 2)*

*Vul het best passende antwoord in.*

| Helemaal Oneens | Oneens | Neutraal | Eens | Helemaal Eens |
| --- | --- | --- | --- | --- |
| 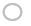 | 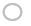 | 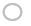 | 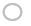 | 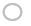 |

***Need for Recovery****

*Introduction to items**:

|  | Nooit | Soms | Vaak | Altijd |
| --- | --- | --- | --- | --- |
| NFR Item 1 | 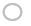 | 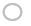 |  |  |
| NFR Item 2 | 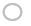 | 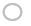 | 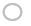 | 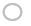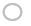 |
| NFR Item 3 | 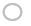 | 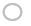 | 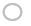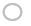 | 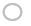 |
| NFR Item 4 | 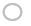 | 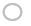 | 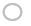 | 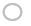 |
| NFR Item 5 | 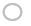 | 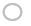 | 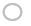 | 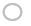 |
| NFR Item 6 | 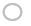 | 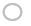 | 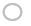 | 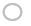 |

*Note: due to copy rights, the items of the Dutch version of the Need for Recovery Scale [26] are not shown.

***Motivation****

*Introduction to items**:

|  | Nooit | Een paar keer per jaar of minder | Eens per maand of minder | Een paar keer per maand | Eens per week | Een paar keer per week | Dagelijks |
| --- | --- | --- | --- | --- | --- | --- | --- |
| UWES-1 *(Work-engagement, vigour)* | 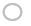 | 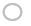 |  |  | 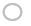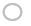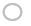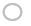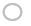 |  |  |
| UWES-2 *(Work-engagement, dedication)* |  |  |  |  |  |  | 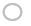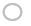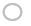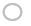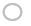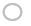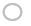 |
| UWES-3*(Work-engagement, absorption)* | 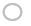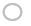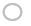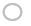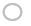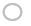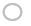 |  |  |  |  |  |  |

*Note: due to copy rights, the items of the UWES-3 scale [37] are not shown

***Job Satisfaction***

Over het algemeen ben ik tevreden met mijn werk. *(Job satisfaction)*

*Vul het best passende antwoord in.*

| Helemaal Oneens | Oneens | Neutraal | Eens | Helemaal Eens |
| --- | --- | --- | --- | --- |
| 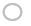 | 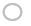 | 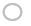 | 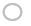 | 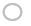 |

***Skill gap***

De eisen van mijn baan en wat ik kan, zijn op elkaar afgestemd. *(Demands-Abilities 1)*

*Vul het best passende antwoord in.*

| Helemaal Oneens | Oneens | Neutraal | Eens | Helemaal Eens |
| --- | --- | --- | --- | --- |
| 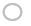 | 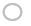 | 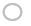 | 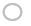 | 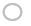 |

Wat ik kan en mijn opleiding passen bij de eisen van de baan. *(Demands-Abilities 2)*
*Vul het best passende antwoord in.*

| Helemaal Oneens | Oneens | Neutraal | Eens | Helemaal Eens |
| --- | --- | --- | --- | --- |
| 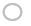 | 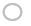 | 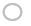 | 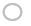 | 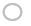 |

Wat ik kan en mijn opleiding passen goed bij wat het werk van mij vraagt. *(Demands-Abilities 3)*

*Vul het best passende antwoord in.*

| Helemaal Oneens | Oneens | Neutraal | Eens | Helemaal Eens |
| --- | --- | --- | --- | --- |
| 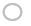 | 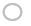 | 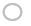 | 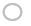 | 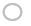 |

***Performance***

Ik vervul de verantwoordelijkheden die horen bij mijn baan. (*Performance 1)*

*Vul het best passende antwoord in.*

| Helemaal Oneens | Oneens | Neutraal | Eens | Helemaal Eens |
| --- | --- | --- | --- | --- |
| 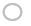 | 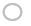 | 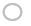 | 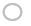 | 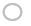 |

Ik voer de taken uit die van mij verwacht worden. (*Performance 2)*

*Vul het best passende antwoord in.*

| Helemaal Oneens | Oneens | Neutraal | Eens | Helemaal Eens |
| --- | --- | --- | --- | --- |
| 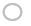 | 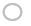 | 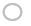 | 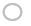 | 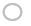 |

Ik doe mijn werk goed. (*Performance 3)*

*Vul het best passende antwoord in.*

| Helemaal Oneens | Oneens | Neutraal | Eens | Helemaal Eens |
| --- | --- | --- | --- | --- |
| 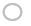 | 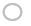 | 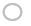 | 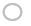 | 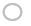 |

De werkbegeleider is tevreden over mijn werk. (*performance 4)*

*Vul het best passende antwoord in.*

| Helemaal Oneens | Oneens | Neutraal | Eens | Helemaal Eens |
| --- | --- | --- | --- | --- |
| 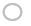 | 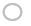 | 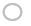 | 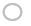 | 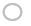 |

***Employability***

Ik vertrouw erop dat ik mijn huidige baan kan houden. (*Internal Employability)*

*Vul het best passende antwoord in.*

| Helemaal Oneens | Oneens | Neutraal | Eens | Helemaal Eens |
| --- | --- | --- | --- | --- |
|  |  |  |  |  |

Ik vertrouw erop dat ik een andere baan vind als ik zou zoeken. (*External employability 1*)

*Vul het best passende antwoord in.*

| Helemaal Oneens | Oneens | Neutraal | Eens | Helemaal Eens |
| --- | --- | --- | --- | --- |
|  |  |  |  |  |

Het zal voor mij moeilijk zijn om een andere baan te vinden als ik [*name of organisation*] verlaat. (*External employability 2)*

*Vul het best passende antwoord in.*

| Helemaal Oneens | Oneens | Neutraal | Eens | Helemaal Eens |
| --- | --- | --- | --- | --- |
|  |  |  |  |  |

Als ik ontslagen wordt, zal ik gemakkelijk een gelijkwaardige baan vinden. (*External employability 3)*

*Vul het best passende antwoord in.*

| Helemaal Oneens | Oneens | Neutraal | Eens | Helemaal Eens |
| --- | --- | --- | --- | --- |
|  |  |  |  |  |

# Work-related Sense of Coherence

Ik kan mijn baan niet aan (*WSC1co_r*)

*Vul het best passende antwoord in.*

| Helemaal Oneens | Oneens | Neutraal | Eens | Helemaal Eens |
| --- | --- | --- | --- | --- |
|  |  |  |  |  |

Ik vind mijn baan betekenisvol. (*WSC2me*)

*Vul het best passende antwoord in.*

| Helemaal Oneens | Oneens | Neutraal | Eens | Helemaal Eens |
| --- | --- | --- | --- | --- |
|  |  |  |  |  |

Ik vind mijn baan ongestructureerd. (*WSC3co_r*)

*Vul het best passende antwoord in.*

| Helemaal Oneens | Oneens | Neutraal | Eens | Helemaal Eens |
| --- | --- | --- | --- | --- |
|  |  |  |  |  |

Ik kan mijn baan niet beïnvloeden (*WSC4ma_r*)

*Vul het best passende antwoord in.*

| Helemaal Oneens | Oneens | Neutraal | Eens | Helemaal Eens |
| --- | --- | --- | --- | --- |
|  |  |  |  |  |

Ik vind mijn baan belangrijk. (*WSC5me*)

*Vul het best passende antwoord in.*

| Helemaal Oneens | Oneens | Neutraal | Eens | Helemaal Eens |
| --- | --- | --- | --- | --- |
|  |  |  |  |  |

Ik vind mijn taken niet duidelijk. (*WSC6co_r*)

*Vul het best passende antwoord in.*

| Helemaal Oneens | Oneens | Neutraal | Eens | Helemaal Eens |
| --- | --- | --- | --- | --- |
|  |  |  |  |  |

Ik heb geen controle over mijn werk. (*WSC7ma_3*)

*Vul het best passende antwoord in.*

| Helemaal Oneens | Oneens | Neutraal | Eens | Helemaal Eens |
| --- | --- | --- | --- | --- |
|  |  |  |  |  |

Ik vind mijn baan de moeite waard. (*WSC8me*)

*Vul het best passende antwoord in.*

| Helemaal Oneens | Oneens | Neutraal | Eens | Helemaal Eens |
| --- | --- | --- | --- | --- |
|  |  |  |  |  |

Ik vind mijn baan niet voorspelbaar. (*WSC9co_r*)

*Vul het best passende antwoord in.*

| Helemaal Oneens | Oneens | Neutraal | Eens | Helemaal Eens |
| --- | --- | --- | --- | --- |
|  |  |  |  |  |

# Person-Job Fit

Mijn baan sluit goed aan op wat ik zoek in een baan. *(Needs-supplies fit 1)*

*Vul het best passende antwoord in.*

| Helemaal Oneens | Oneens | Neutraal | Eens | Helemaal Eens |
| --- | --- | --- | --- | --- |
|  |  |  |  |  |

De eisen van mijn baan en wat ik kan, zijn op elkaar afgestemd. *(Demands-Abilities 1)*

*Vul het best passende antwoord in.*

| Helemaal Oneens | Oneens | Neutraal | Eens | Helemaal Eens |
| --- | --- | --- | --- | --- |
|  |  |  |  |  |

Mijn baan heeft de eigenschappen die ik zoek in een baan. *(Needs-supplies fit 2)*

*Vul het best passende antwoord in.*

| Helemaal Oneens | Oneens | Neutraal | Eens | Helemaal Eens |
| --- | --- | --- | --- | --- |
|  |  |  |  |  |

Wat ik kan en mijn opleiding passen bij de eisen van de baan. *(Demands-Abilities 2)*
*Vul het best passende antwoord in.*

| Helemaal Oneens | Oneens | Neutraal | Eens | Helemaal Eens |
| --- | --- | --- | --- | --- |
|  |  |  |  |  |

Mijn baan biedt mij waar ik behoefte aan heb in een baan. *(Needs-supplies fit 3)*

*Vul het best passende antwoord in.*

| Helemaal Oneens | Oneens | Neutraal | Eens | Helemaal Eens |
| --- | --- | --- | --- | --- |
|  |  |  |  |  |

Wat ik kan en mijn opleiding passen goed bij wat het werk van mij vraagt. *(Demands-Abilities 3)*

*Vul het best passende antwoord in.*

| Helemaal Oneens | Oneens | Neutraal | Eens | Helemaal Eens |
| --- | --- | --- | --- | --- |
|  |  |  |  |  |
