# Supplementary material for: Sustainable Employability of People with Limited Capability for Work: The Participatory Development and Validation of a Questionnaire
Source: J Occup Rehabil. 2024 May 20;35(1):105–15. doi: 10.1007/s10926-024-10191-1 (PMC11839800; doi:10.1007/s10926-024-10191-1)
Supplement: Supplementary file 2 — Supplementary file2 (DOCX 116 KB) [file 10926_2024_10191_MOESM2_ESM.docx]

# Supplementary information B

**Article title:** Sustainable Employability of People with Limited Capability for Work: the Participatory Development and Validation of a Questionnaire

**Journal:** Journal of Occupational Rehabilitation

**Authors:** S.R Hiemstra, B.P.I. Fleuren, A. de Jonge, J. Naaldenberg & L. Vaandrager

**Corresponding Author:** S.R. Hiemstra

**Introduction**

This electronic supplement provides information on (A) factor loadings of individual items for the used multiple-item (≥ 3 items) scales to measure SE, PJ-Fit and Work-SoC for people with OD, (B) an exploration of the hypothesized factor model for SE for people with Limited Capability for Work (LCW), and a competing model and it’s factor loadings as referred to in the article.

# A. Factor loadings of individual items of the developed questionnaire

Table 1-6 provide insights in the item-factor loadings for each (adjusted) multiple-item scale to measure indicators of SE and the scales for PJ- Fit and Work-SoC.

**Table 1.** Item-factor loadings for the Need for Recovery Scale* [26]

| Item | Loading | Standard Error | Est./S.E. | P-value |
| --- | --- | --- | --- | --- |
| NFR item 1 | 0.744 | 0.037 | 20.035 | *p <*.001 |
| NFR item 2 | 0.866 | 0.034 | 25.239 | *p <*.001 |
| NFR item 3 | 0.782 | 0.038 | 20.366 | *p <*.001 |
| NFR item 4 | 0.735 | 0.041 | 17.908 | *p <*.001 |
| NFR item 5 | 0.799 | 0.034 | 23.619 | *p <*.001 |
| NFR item 6 | 0.656 | 0.041 | 15.828 | *p <*.001 |

*Note: Reported factor loadings are standardised, transcribed from Mplus7 STDYX Output.*

** = Due to copyright, the items are not shown.*

**Table 2.** Item-factor loadings for Employability, adapted from Janssens, Sels & Van den Brande [28]

| Item | Loading | Standard Error | Est./S.E. | P-value |
| --- | --- | --- | --- | --- |
| I am confident that I would find another job if I were to look for it (External employability 1) | 1.000 | 0.000 | 999.000** | N/a |
| It will be difficult for me to find another job if I leave [organization] (reversed) (External employability 2) | 0.636 | 0.036 | 17.924 | *p <*.001 |
| If I would be fired, I will easily find another job (External Employability 3) | 0.653 | 0.038 | 17.121 | *p <*.001 |

*Note: Reported factor loadings are standardised, transcribed from Mplus7 STDYX Output, ** = Factor variance was fixed to 1 to circumvent model saturation.*

**Table 3.** Item-factor loadings for Performance, adapted from the Core Task Performance Scale [12, 29]

| Item | Loading | Standard Error | Est./S.E. | P-value |
| --- | --- | --- | --- | --- |
| I fulfil the tasks that are part of my job (Performance 1) | 0.735 | 0.038 | 19.398 | *p <*.001 |
| I perform the tasks that are expected of me (Performance 2) | 0.913 | 0.023 | 40.192 | *p <*.001 |
| I do my work well (Performance 3) | 0.847 | 0.025 | 33.373 | *p <*.001 |
| My supervisor is satisfied with my work (Performance 4) | 0.801 | 0.031 | 26.093 | *p <*.001 |

*Note: Reported factor loadings are standardised, transcribed from Mplus7 STDYX Output*

**Table 4.** Item-factor loadings for UWES-3* [37]

| Item | Loading | Standard Error | Est./S.E. | P-value |
| --- | --- | --- | --- | --- |
| UWES-1 (Work-engagement, vigour) | 1.000 | 0.000 | 999.000** | N/a |
| UWES-2 (Work-engagement, dedication) | 0.838 | 0.028 | 30.466 | *p <*.001 |
| UWES-3 (Work-engagement, absorption) | 0.773 | 0.032 | 24.195 | *p <*.001 |

*Note: Reported factor loadings are standardised, transcribed from Mplus7 STDYX Output, ** = Factor variance was fixed to 1 to circumvent model saturation. * = Due to copyright, the items are not shown.*

**Table 5.** Item-factor loadings for Person-Job Fit, adapted from Person-Job Fit Scale [27]

| Item | Loading | Standard Error | Est./S.E. | P-value |
| --- | --- | --- | --- | --- |
| Needs-Supplies Fit |  |  |  |  |
| My job matches with what I am looking for in a job (Needs-Supplies 1) | 0.949 | 0.013 | 75.850 | *p <*.001 |
| My job has the characteristics I am looking for in a job (Needs-Supplies 2) | 0.896 | 0.015 | 58.549 | *p <*.001 |
| My job provides me with what I need in a job (Needs-Supplies 3) | 0.878 | 0.022 | 39.899 | *p <*.001 |
|  |  |  |  |  |
| Demands-Abilities Fit |  |  |  |  |
| The requirements of my job and what I can do are attuned to each other (Demands-Abilities 1) | 0.799 | 0.027 | 29.922 | *p <*.001 |
| My abilities and my education match the requirements of the job (Demands-Abilities 2) | 0.840 | 0.023 | 36.819 | *p <*.001 |
| My abilities do and my education fit well with what the job requires of me (Demands-Abilities 3) | 0.867 | 0.024 | 35.814 | *p <*.001 |

*Note: Reported factor loadings are standardised, transcribed from Mplus7 STDYX Output*

## **Table 6.** Item-factor loadings for Work-SoC, adapted from Work-SoC scale [19]

| Item | Loading | Standard Error | Est./S.E. | P-value |
| --- | --- | --- | --- | --- |
| Comprehensibility |  |  |  |  |
| I can’t handle my work (reversed) (WSC1co_r) | 0.663 | 0.058 | 11.428 | *p <*.001 |
| I find my job unstructured (reversed) (WSC3co_r) | 0.636 | 0.047 | 13.410 | *p <*.001 |
| I find my tasks unclear (reversed) (WSC6co_r) | 0.700 | 0.054 | 13.003 | *p <*.001 |
| I find my work not predictable (reversed) (WSC9_r) | 0.328 | 0.057 | 5.742 | *p <*.001 |
|  |  |  |  |  |
| Meaningfulness: |  |  |  |  |
| I find my job meaningful (WSC2me) | 0.730 | 0.036 | 20.318 | *p <*.001 |
| I find my job important (WSC5me) | 0.918 | 0.018 | 51.170 | *p <*.001 |
| I find my job worthwhile (WSC8me) | 0.859 | 0.024 | 36.146 | *p <*.001 |
|  |  |  |  |  |
| Manageability: |  |  |  |  |
| I cannot influence my job (reversed) (WSC4ma_r) | 0.477 | 0.053 | 9.065 | *p <*.001 |
| I have no control over my work (reversed) (WSC7ma_r) | 0.882 | 0.057 | 15.432 | *p <*.001 |

*Note: Reported factor loadings are standardised, transcribed from Mplus7 STDYX Output*

# B. Exploration of (competing) factor models for SE

The initial questionnaire was based on the hypothesis that SE could be measured as a formative construct, with the eight used indicators for SE. To check this hypothesis and to due to insights in the relevance of specific items (i.e. with regards to internal mobility), the authors were motivated to check a competitive model: Model 2, in which the item for internal employability was excluded. Results of the two conducted CFA’s can be found in table 7. Further explanations of the models and are described in this Supplement.

**Table 7**. Exploration of competing models

| First-order factor  (*n* = *248*) | χ2 | Df | CFI | TLI | RMSEA | RMSEA 90% C.I. | Range factor loadings items |
| --- | --- | --- | --- | --- | --- | --- | --- |
| Model 1. Eight-factor model SE  *(all hypothesised indicator items)* | 424.008* | 222 | 0.910 | 0.888 | 0.061 | 0.052 - 0.069 | 0.542 – 0.928 |
| 2. Eight-factor model SE  *(without internal employability item)* | 351.914* | 205 | 0.931 | 0.915 | 0.054 | 0.044 - 0.063 | 0.560 – 0.922 |

*Note: ML estimator was used. Transcribed from Mplus7 Output*

**Model 1: Hypothesised eight-factor model**First, we conducted a CFA that tested the hypothesised model of SE (Model 1) as formative construct with 8 SE indicators, based on the adjusted items for the proposed indicator scales. This led to an overall fit (CFI = .91; TLI= .89; RMSEA = .061 [CI90% .052 - .069], see table 7). Factor loadings of this model proved to be adequate (see table 8).

**Figure 2:** Visual representation Model 1 – Eight-factor model for SE.
The model contains formative indicators for SE. Oval represents latent (unobserved) variable, square represent observed variables as estimated from scale items.

**Table 8** Factor loadings Model 1. Hypothesised eight-factor model of SE

| Factor  and items | Loading | Standard Error | Est./S.E. | P-value |
| --- | --- | --- | --- | --- |
| Perceived health |  |  |  |  |
| In general, would you say your health is… (perceived health) | 1.000 | 0.000 | 999.000^a^ | N/a |
| Need for Recovery (reverse coded for overall SE)* |  |  |  |  |
| NFR item 1 | 0.656 | 0.044 | 15.029 | *p <*.001 |
| NFR item 2 | 0.745 | 0.039 | 19.329 | *p <*.001 |
| NFR item 3 | 0.671 | 0.044 | 15.248 | *p <*.001 |
| NFR item 4 | 0.635 | 0.045 | 13.960 | *p <*.001 |
| NFR item 5 | 0.683 | 0.043 | 15.812 | *p <*.001 |
| NFR item 6 | 0.584 | 0.050 | 11.701 | *p <*.001 |
| Workability |  |  |  |  |
| In general, I feel physically healthy enough for work (Workability 1) | 0.706 | 0.045 | 15.715 | *p <*.001 |
| In general, I feel mentally healthy enough for work (Workability 2) | 0.726 | 0.044 | 16.546 | *p <*.001 |
| Skill gap |  |  |  |  |
| The requirements of my job and what I can do are attuned to each other (Demands-Abilities 1) | 0.542 | 0.049 | 11.058 | *p <*.001 |
| My abilities and my education match the requirements of the job (Demands-Abilities 2) | 0.817 | 0.030 | 27.608 | *p <*.001 |
| My abilities do and my education fit well with what the job requires of me (Demands-Abilities 3) | 0.888 | 0.028 | 31.495 | *p <*.001 |
| Employability |  |  |  |  |
| I am confident that I can keep my current job (Internal employability) | 1.000 | 0.000 | 999.000^a^ | N/a |
| I am confident that I would find another job if I were to look for it (External employability 1) | 0.735 | 0.048 | 15.371 | *p <*.001 |
| It will be difficult for me to find another job if I leave [organization] (reversed) (External employability 2) | 0.702 | 0.050 | 13.975 | *p <*.001 |
| If I would be fired, I will easily find another job (External Employability 3) | 0.698 | 0.049 | 14.230 | *p <*.001 |
| Performance |  |  |  |  |
| I fulfill the tasks that are part of my job (Performance 1) | 0.620 | 0.044 | 14.012 | *p <*.001 |
| I perform the tasks that are expected of me (Performance 2) | 0.772 | 0.034 | 22.424 | *p <*.001 |
| I do my work well (Performance 3) | 0.765 | 0.034 |  | *p <*.001 |
| My supervisor is satisfied with my work (Performance 4) | 0.734 | 0.036 | 20.350 | *p <*.001 |
| Motivation* |  |  |  |  |
| UWES-1 (Work-engagement, vigor) | 0.620 | 0.046 | 13.514 | *p <*.001 |
| UWES-2 (Work-engagement, dedication) | 0.928 | 0.029 | 32.268 | *p <*.001 |
| UWES-3(Work-engagement, absorbation) | 0.729 | 0.038 | 19.299 | *p <*.001 |
| Job satisfaction |  |  |  |  |
| In general, I am satisfied with my job (Job satisfaction) | 1.000 | 0.000 | 999.000^a^ | N/a |

*Note: ML estimator was used. Transcribed from Mplus7 Output. Reported factor loadings are standardised, transcribed from Mplus7 STDYX Output; * = Due to copyright, the items are not shown; ^a^ = conventional value for single items using Mplus 7.*

**Model 2 – Eight-factor model without internal employability.**

During the cognitive interviews, it became clear that in the specific context of this research, internal employability could rather be an indicator of organizational policy than internal employability of the individual. The organization that cooperated in this study, emerged from national policy to support employment for people with OD. This employer specifically creates jobs for this target group and aims for long term employment and job security for people with OD, by providing a permanent contract when people are performing well and are content with their job. Therefore, a CFA was done to check the model fit when this indicator was left out of our model. This led to a better, good fit of the model (CFI = .93; TLI= .92; RMSEA = .054 [CI90% .044 - .063], see Table 7). Factor loadings of this model proved to be adequate (see Table 9).

**Figure 3:** Visual representation Model 2– Eight-factor model for SE without internal employability.
The model contains formative indicators for SE. Oval represents latent (unobserved) variable, square represent observed variables. Internal employability is excluded in Model 2

**Table 9** Factor loadings Model 2. Eight-factor model of SE without item for Internal Employability

| Factor  and items | Loading | Standard Error | Est./S.E. | P-value |
| --- | --- | --- | --- | --- |
| Perceived health |  |  |  |  |
| In general, would you say your health is… (perceived health) | 1.000 | 0.000 | 999.000^a^ | N/a |
| Need for Recovery (reverse coded for overall SE)* |  |  |  |  |
| NFR item 1 | 0.659 | 0.043 | 15.159 | *p <*.001 |
| NFR item 2 | 0.749 | 0.038 | 19.668 | *p <*.001 |
| NFR item 3 | 0.675 | 0.044 | 15.434 | *p <*.001 |
| NFR item 4 | 0.638 | 0.045 | 14.027 | *p <*.001 |
| NFR item 5 | 0.686 | 0.043 | 15.897 | *p <*.001 |
| NFR item 6 | 0.587 | 0.050 | 11.732 | *p <*.001 |
| Workability |  |  |  |  |
| In general, I feel physically healthy enough for work (Workability 1) | 0.721 | 0.043 | 16.648 | *p <*.001 |
| In general, I feel mentally healthy enough for work (Workability 2) | 0.736 | 0.043 | 17.180 | *p <*.001 |
| Skill gap |  |  |  |  |
| The requirements of my job and what I can do are attuned to each other (Demands-Abilities 1) | 0.560 | 0.050 | 11.225 | *p <*.001 |
| My abilities and my education match the requirements of the job (Demands-Abilities 2) | 0.827 | 0.029 | 28.562 | *p <*.001 |
| My abilities do and my education fit well with what the job requires of me (Demands-Abilities 3) | 0.896 | 0.027 | 32.862 | *p <*.001 |
| Employability |  |  |  |  |
| I am confident that I would find another job if I were to look for it (External employability 1) | 0.731 | 0.048 | 15.335 | *p <*.001 |
| It will be difficult for me to find another job if I leave [organization] (reversed) (External employability 2) | 0.703 | 0.050 | 14.022 | *p <*.001 |
| If I would be fired, I will easily find another job (External Employability 3) | 0.697 | 0.049 | 14.171 | *p <*.001 |
| Performance |  |  |  |  |
| I fulfil the tasks that are part of my job (Performance 1) | 0.645 | 0.044 | 14.796 | *p <*.001 |
| I perform the tasks that are expected of me (Performance 2) | 0.802 | 0.033 | 24.619 | *p <*.001 |
| I do my work well (Performance 3) | 0.782 | 0.033 | 23.416 | *p <*.001 |
| My supervisor is satisfied with my work (Performance 4) | 0.723 | 0.037 | 19.392 | *p <*.001 |
| Motivation* |  |  |  |  |
| UWES-1 (Work-engagement, vigour) | 0.617 | 0.046 | 13.486 | *p <*.001 |
| UWES-2 (Work-engagement, dedication) | 0.922 | 0.029 | 31.433 | *p <*.001 |
| UWES-3 (Work-engagement, absorption) | 0.725 | 0.038 | 19.238 | *p <*.001 |
| Job satisfaction |  |  |  |  |
| In general, I am satisfied with my job (Job satisfaction) | 1.000 | 0.000 | 999.000^a^ | N/a |

*Note: ML estimator was used. Transcribed from Mplus7 Output. Reported factor loadings are standardised, transcribed from Mplus7 STDYX Output; * = Due to copyright, the items are not shown; ^a^ = conventional value for single items using Mplus 7.*
